# Model-informed drug development of novel ROCK2 inhibitor TDI01: population pharmacokinetic study and simulation

**DOI:** 10.3389/fphar.2025.1477607

**Published:** 2025-03-04

**Authors:** Xiwei Ji, Yimin Cui

**Affiliations:** Institute of Clinical Pharmacology, Peking University First Hospital, Beijing, China

**Keywords:** Pop-PK, TDI01, hepato-enteral circulation, model-informed drug development, simulation

## Abstract

**Introduction:**

TDI01 is a novel and highly selective ROCK2 inhibitor, which provides a potential valuable candidate for the treatment of ALI/ARDS due to COVID-19.

**Methods:**

The objective of this study was to develop Pop-PK models to characterize the PK properties of TDI, and to guide the choice of suitable clinical dosage regimens via model-based simulation.

**Results:**

The completed clinical study suggested a double peak phenomenon, which could be observed after single administration of TDI01. Thus, a one-compartment model with dosage effect and hepato-enteral circulation were developed to describe the PK profiles of TDI01 in vivo. Results from the simulation show that the drug accumulation observed after multiple doses would not affect the safety of TID01 usage under the tested dosage regimen.

**Discussion:**

The established model and simulation provide a useful approach to maximize the clinical medication safety and therapeutic efficacy of TDI01.

## Introduction

TDI01 is a novel ROCK2 inhibitor that has been tested in clinical trials for the treatment of idiopathic pulmonary fibrosis and silicosis. The study results suggested that TDI01 protected against inflammation and vascular leakage in LPS-induced acute lung injury/acute respiratory distress syndrome (ALI/ARDS) caused by mild, moderate to severe coronavirus disease 2019 (COVID-19) (Hu, Guo, Zhou and Shi, 2021). Additionally, TDI01 can rescue endothelial dysfunction by reducing the expression of VCAM1 and ICAM1, weakening inflammatory cell adhesion and attenuating permeability (Deng et al., 2023).

Model-informed drug development (MIDD) is considered as an important component of modern drug development, which applies various models derived from preclinical and clinical data sources to address drug development or promote decision-making process (Marshall et al., 2016; Venkatakrishnan et al., 2015; Wilkins et al., 2017). In this study, the MIDD approaches are utilized with the aim to optimize benefit–risk and improving TDI01 development efficiency.

The purpose of this study was to develop Pop-PK models to characterize the PK properties of TDI, and to guide the choice of clinical dosage regimens by model-based simulation. Ultimately, the PK modeling and simulation may provide a feasible approach to assure the clinical medication safety.

## Development of population pharmacokinetics models

### Human PK data collection

PK data from the clinical phase I study (clinical trial approval of TDI01 was obtained from Beijing Tide Pharmaceutical Co., Ltd. Study participants received single-dose or multiple-dose oral administration of TDI01. The single-dose regimens included: 400 mg QD, 800 mg QD, and 1200 mg QD; the multiple-dose regimens included: 200 mg QD, 7 days and 400 mg QD, 7 days. Blood samples in single dose groups were collected prior to drug administration (0 h) and at 1, 2, 3, 3.5, 4, 5, 6, 8, 10, 12, 24, 36, 48, and 72 h post administration. Blood samples in multiple dose groups were collected at 0, 1, 2, 3, 3.5, 4, 5, 6, 8, 10, 12, 24, 48, 72, 96, 120, 144, 145, 146, 147, 147.5, 148, 149, 150, 152, 154, 156, 168, 180, 192, and 216 h.

### Structural model

Conventional compartmental PK models were tested sequentially. Model selection was based on changes in the Akaike information criterion (AIC), the precision of parameter estimates (relative standard errors, RSE) and standard goodness-of-fit (GoF) plots (Lindbom, Pihlgren and Jonsson, 2005).

One-compartment, two-compartment and hepato-enteral circulation models were investigated. After comparing the AIC and the model-fitting degree, optimal model was utilized as the structural model. The covariates known with significant impact were also included in the structural model firstly. The First-order conditional estimation with interaction (FOCEI) was used to fit the parameters in this analysis.

### Random effects model

The random effects model included inter-individual random effects and residual random effects. An exponential model (Equation 1) was used to describe inter-individual variation, shown as follows:

$$P_{i}=\theta \cdot \exp\left(\eta_{i}\right)$$ (1)

where P_i_ is the PK parameter of each individual, θ is the PK parameter of the population and η_i_ represent the inter individual variation which follows a logarithmic normal distribution. A combined error model (Equation 2) was used to describe residual error:

$$Y_{ij}=C_{ij}\cdot \left(1+\varepsilon_{1ij}\right)+\varepsilon_{2ij},$$ (2)

where, $Y_{ij}$ represents the observed values, $Y_{ij}$ represents the population predicted values and, ε_1_ and ε_2_ represent additive and proportional residual error, respectively.

### Model evaluation

The final PK model was evaluated graphically on GoF plots, including observed values versus individual prediction or population prediction, conditional weighted residuals (CWRES) versus TIME, absolute individual weighted residuals (|IWRES|) versus individual predictions, and the normality test of CWRES.

Bootstrap was performed to internally validate the final model. The original dataset was used to simulate 1,000 additional datasets, each of which was used to re-estimate the parameters with the final model. Median values and 95% confidence intervals (CI) were calculated and compared with the final model parameter estimates to assess the robustness of the final model.

Visual predictive checks (VPCs) were used to evaluate the predictive power of the final model. One thousand simulations were implemented and the 2.5th, 50th and 97.5th percentiles of the observed versus simulated data were compared.

### Handling of missing, below-quantitative-limit (BQL), and outlier data

Analyte concentrations that were below the lower limit of quantification (LLOQ) were reported as data below the quantification limit (BQL). If the proportion of BQL data was larger than 10%, the likelihood-based approach (such as the M1 method) along with Laplacian estimation was used (Ahn et al., 2008; Beal, 2001). Observations corresponded to absolute conditional weighted residuals of more than 5 (|CWRES| > 5) were regarded as outliers and omitted from the model-building process. These omitted points were re-introduced into the dataset and model evaluation at a later stage (sensitivity analysis) was performed to assess their impact on parameter estimates.

### Software details

The population PK model was performed using NONMEM (version 7.5, ICON Development Solutions, Ellicott City, MD, United States). First order conditional estimation with interaction (FOCEI) method was used for parameter estimation (Keizer et al., 2013). R (version 3.5.3) was used for data preparation, graphical analysis, model diagnostics, and statistical summaries. Xpose^®^ (Jonsson and Karlsson, 1999) and Pearl Speaks NONMEM (PsN^®^, version 4.8.0) (Lindbom, et al., 2005; Lindbom et al., 2004) were used for model diagnostics and to facilitate other model development tasks.

## Results

### Pop-PK model of TID01

**FIGURE 1:**
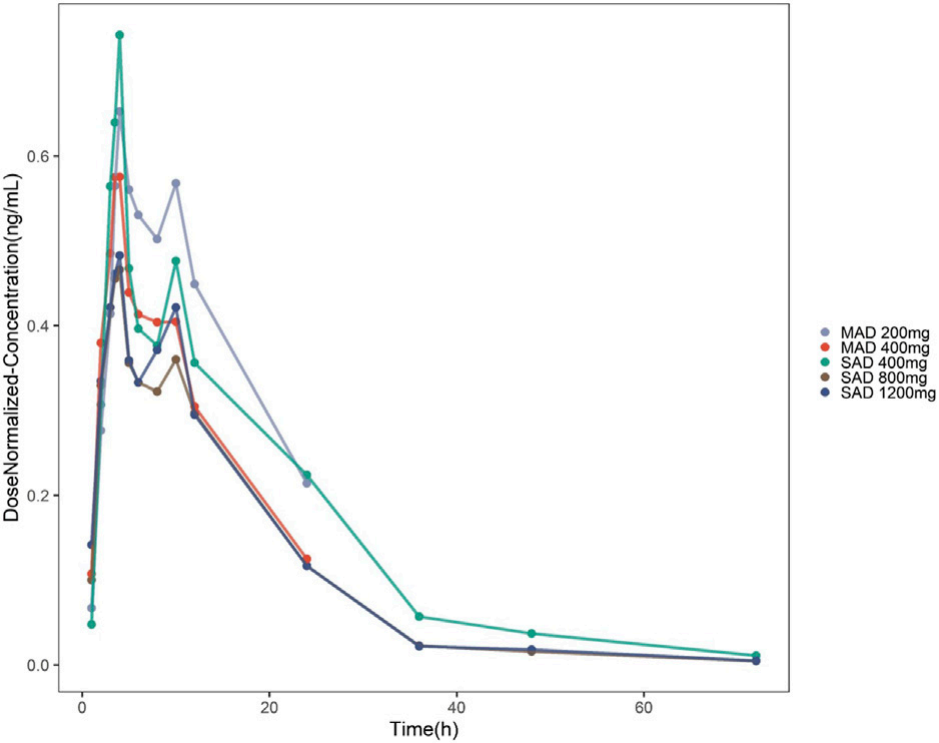
Concentration–time profiles of TDI01 after dose normalization in single-dose and multiple-dose clinical trials (single-dose regimens: 400 mg QD, 800 mg QD and 1,200 mg QD; multiple-dose regimens: 200 mg QD, 7 days and 400 mg QD, 7 days).

In this study, the proportion of BQL data in the overall data is 1.3% (10/786). A total of 776 blood drug concentrations from 39 subjects was included in the data set. As shown in Figure 1, the double peak phenomenon observed after single dosing indicated hepato-enteral circulation model should be included in the final model, and the starting and ending time of hepato-enteral circulation was set as 8–10 h.

**FIGURE 2:**
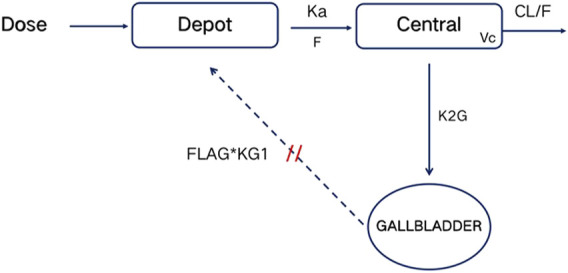
Schematic diagram of PK model. Depot, Central and GALLBLADDER designate the absorption, central and gallbladder compartments, respectively. CL represents clearance rate. K_a_ represents first-order absorption rate constant; K_2G_ represents rate constants for TDI01 from the central compartment to the gallbladder compartment; K_G1_ represents rate constants for TDI01 from the gallbladder compartment to the absorption compartment. FLAG means the time switch of hepato-enteral circulation.

The PK properties of TID01 were described by a one-compartment model with dosage effect and hepato-enteral circulation (Figure 2), which was described by the equations (Equations 3–5) as follows:

K20=CL/Vc

$$\text{DADT}\left(1\right)=\text{FLAG}*K_{G1}*A\left(2\right)-K_{a}*A$$ (3)

$$\text{DADT}\left(2\right)=K_{a}*A-K_{2G}*A\left(1\right)-K_{20}*A\left(1\right)$$ (4)

$$\text{DADT}\left(3\right)=K_{2G}*A\left(1\right)-\text{FLAG}*K_{G1}*A\left(2\right)$$ (5)

where K20 is the clearance rate constant of central compartment; K_a_ represents first-order absorption rate constant; K_2G_ represents rate constants for TDI01 from the central compartment to the gallbladder compartment; K_G1_ represents rate constants for TDI01 from the gallbladder compartment to the absorption compartment. A, A (1) and A (2) represent absorption, central and gallbladder compartments, respectively. FLAG means the time switch of hepato-enteral circulation.

**FIGURE 3:**
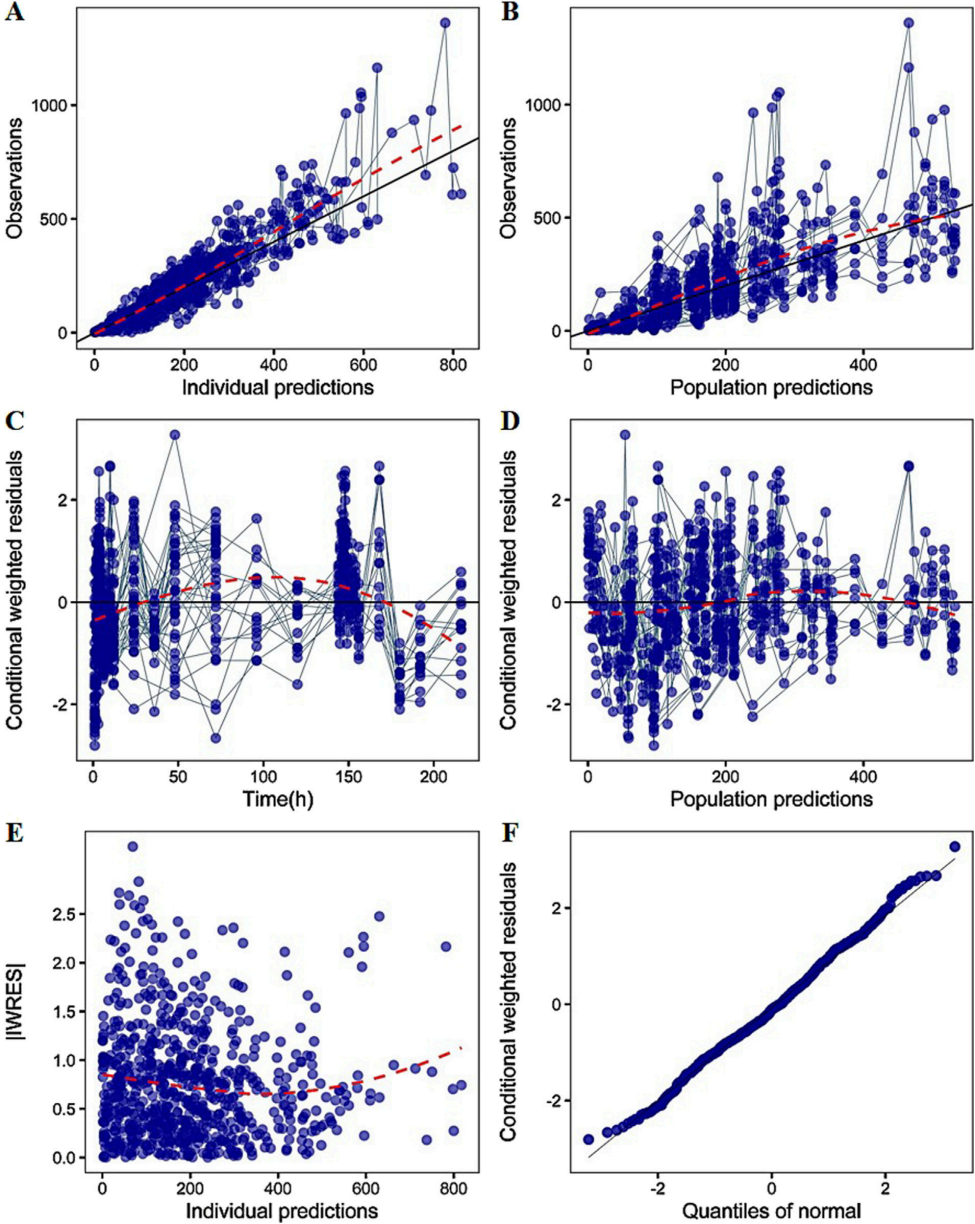
The goodness-of-fit plots of PK model **(A)** Relationship between observed versus IPRED of PK **(B)** Relationship between observed versus PRED of PK **(C)** CWRES at different time points **(D)** CWRES at different PRED **(E)** |IWRES| at different IPRED **(F)** Quantile–quantile (Q–Q) plot of WRES. CWRES: conditional weighted residuals; WRES: weighted residuals; |IWRES|: absolute individual weighted residuals; PRED: predicted value; IPRED: individual predicted value. The solid lines represent the x = y lines. The dotted lines are the trend lines.

**FIGURE 4:**
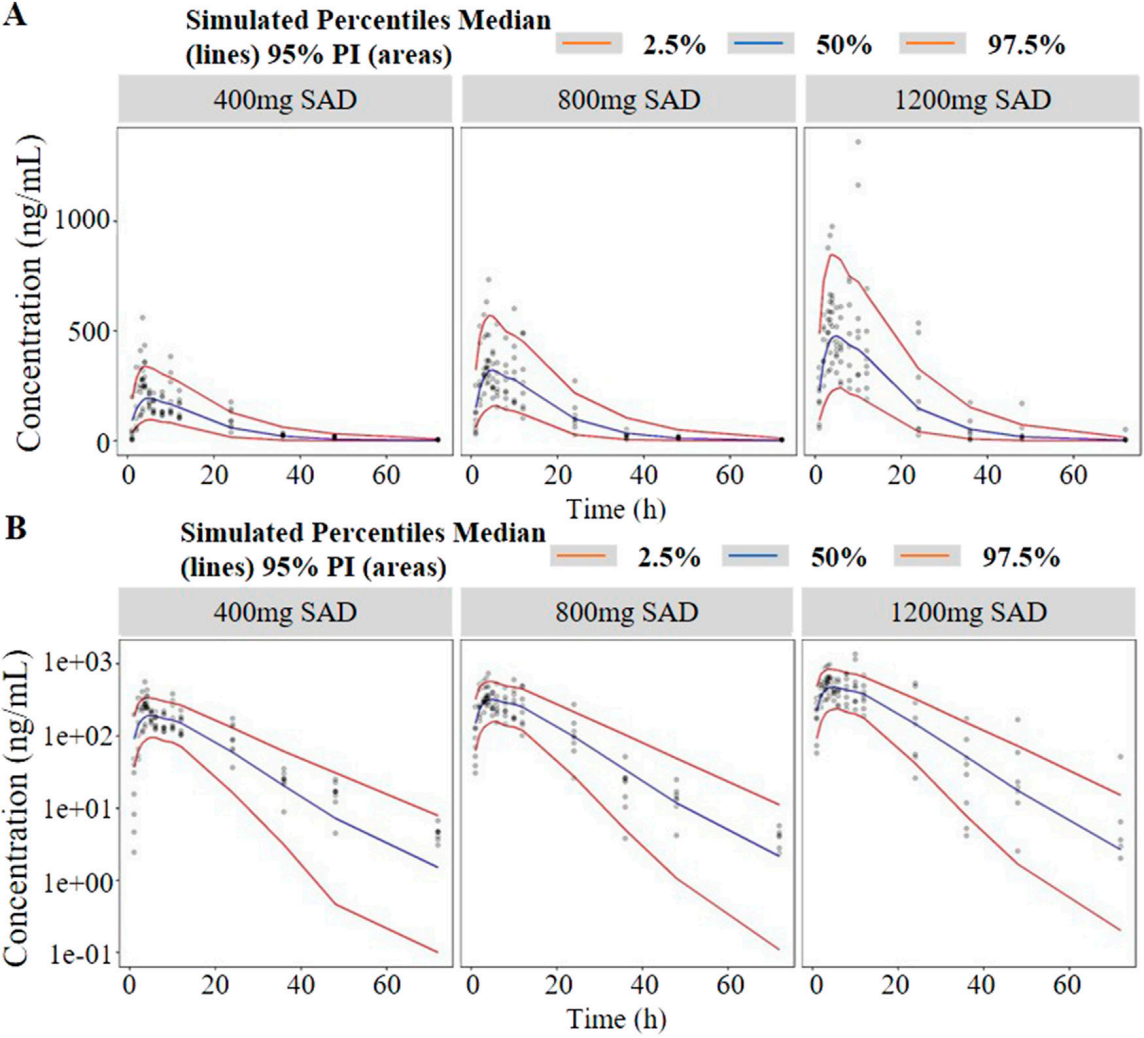
Visual predictive check (VPC) of single-ascending doses (**(A)** in arithmetic scale; **(B)** in logarithmic scale). The red lines represent the 2.5th and 97.5th percentiles of the observed versus simulated data. The range between the red lines depicts the 95th percentile intervals. The blue lines represent the medians of simulated data. Circles represent the observed data.

**FIGURE 5:**
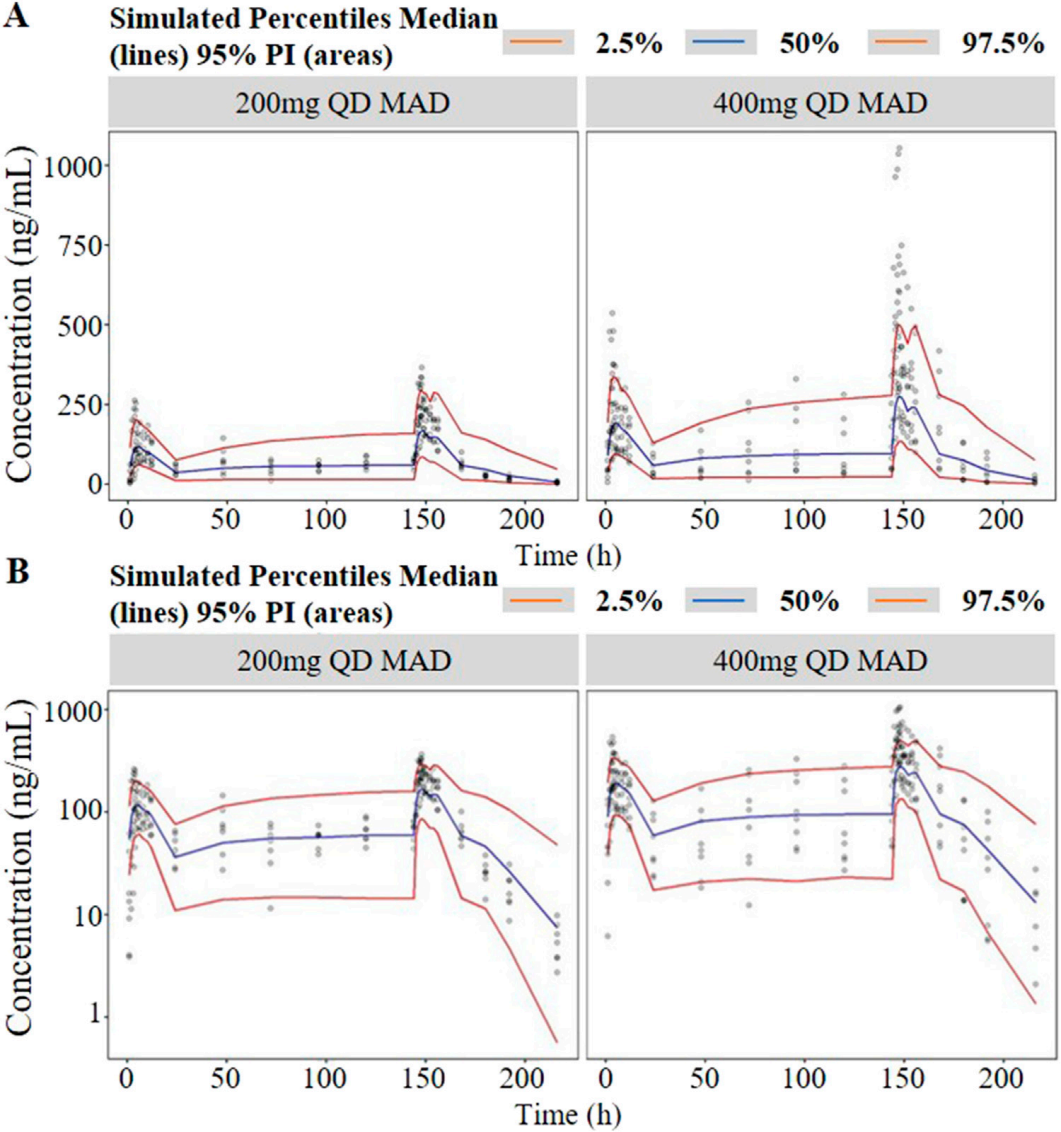
Visual predictive check (VPC) of multiple-ascending doses (**(A)** in arithmetic scale; **(B)** in logarithmic scale). The red lines represent the 2.5th and 97.5th percentiles of the observed versus simulated data. The range between the red lines depicts the 95th percentile intervals. The blue lines represent the medians of simulated data. Circles represent the observed data.

The established model reasonably fit the time courses of TDI01 in the circulatory system with clearance (CL/F) of 95 L/h, volume of distribution (*V*_c_/F) of 1400 L and the absorption rate constant (k_a_/F) of 0.345 h^−1^, respectively (Table 1). As shown in the table, all the model parameters were precisely estimated with relative standard error below 20%. Bootstrap results suggested that the estimated values of the final model parameters were close to the median and within the 95% CI from the non-parametric bootstrap. The goodness-of-fit plots for the developed models are shown in Figure 3. The observed values versus the population and individual predicted values were closely distributed around the line of identity. The conditional weighted residuals were randomly and homogenously distributed around 0. One thousand simulations were implemented and the 2.5th, 50th, and 97.5th percentiles of the observed versus simulated data were compared, as shown in the VPCs of single-ascending doses and multiple-ascending doses (Figures 4, 5). The VPC results for the Pop-PK model indicated that the established models were able to adequately describe the observed plasma concentrations, where most observed plasma concentrations fell within the 95% prediction intervals.

**TABLE 1:** PK parameter and bootstrap of TDI01 final Pop-PK models.

Parameter	Definition	Final model		Bootstrap	
		Estimation	RSE^a^[Shrinkage]^b^	Median	95% CI
CL/F (L/h)	Clearance rate	95	10%	95.15	[82.05–109.52]
V_c_/F (L)	Volume of central compartment	1,400	8%	1,395.22	[1,182.17–1,586.25]
k_a_/F (1/h)	Absorption rate constant	0.345	10%	0.345	[0.264–0.424]
k_2G_/F (1/h)	Inter-compartment transfer rate between central compartment and drug disposal compartment	0.023 (FIX)	—	0.023 (FIX)	—
k_G1_/F (1/h)	Inter-compartment transfer rate between central compartment and gallbladder compartment	2 (FIX)	—	2 (FIX)	—
IIV_CL/F	Individual variation in clearance rate	46.8%	12% [1%]	45.9%	[0.354–0.558]
IIV_V/F	Individual variation in volume of central compartment	32.2%	18% [10%]	31.1%	[0.199–0.411]
IIV_ k_12_	Individual variation in	47.3%	16% [19%]	45.9%	[0.209–0.660]
Prop.error (%)	Proportional residual error	34.2%	3%	34.3%	[0.322–0.363]
Add.error	Additive residual error	1.77	47%	1.795	[1.362–2.271]

^a^ RSE: relative standard error.

^b^ shrinkage values of eta are in the square brackets.

### Prediction of pharmacokinetic profiles of TDI01 by Monte Carlo simulation

**FIGURE 6:**
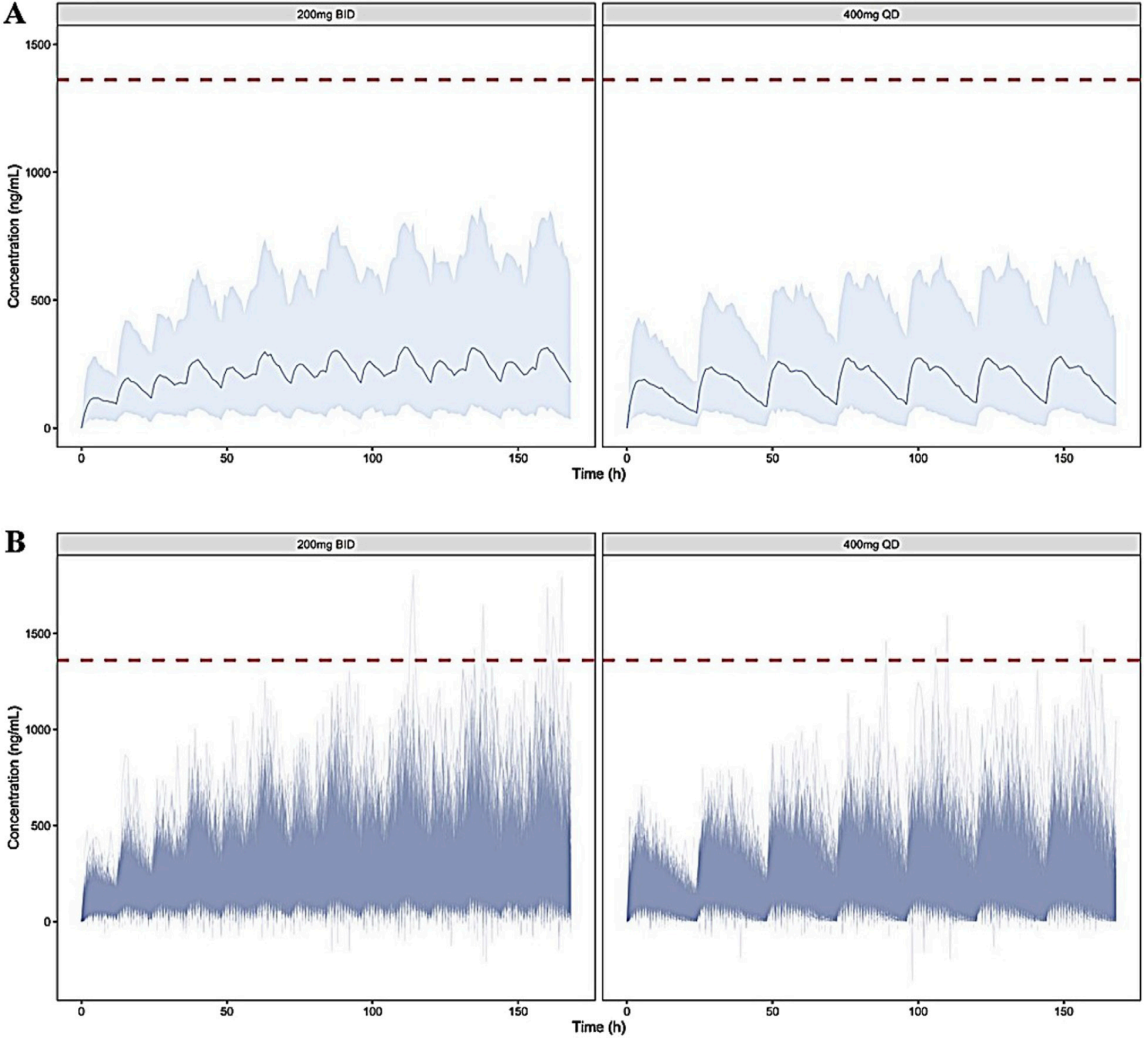
Simulated blood concentration-time profiles of TDI01 under the dosages of 200 mg BID, 7 days and 400 mg QD, 7 days (**(A)** distribution of mean values; **(B)** distribution of individual values). The gray area depicts the 95th percentile intervals. The red dashed lines represent the C_max_ of 1,391 ng/mL under the highest single dose (1,200 mg) in clinical trial.

The simulated concentration-time profiles of TID01 under the designed dose regimens are shown in Figure 6. The regimens are listed in Table 2. The simulated PK parameters are listed in Table 3, which indicated that the C_max_ and AUC values at steady state of 200 mg BID (tested dose regimen) were higher than those of 400 mg QD (dose regimen used in clinical trials), but the differences were less than 25%. As shown in Figure 6, the simulated concentrations of TID01 in blood under the dosages of 200 mg BID, 7 days and 400 mg QD, 7 days are less than the C_max_ of 1,391 ng/mL under the highest single dose (1,200 mg) in clinical trial. The simulation results demonstrated the drug accumulation caused by hepato-enteral circulation would not affect the safety of TID01 usage under the tested multiple dosage regimen.

**TABLE 2:** The regimens of TDI01 used in Monte Carlo simulation.

Dose regimen	Daily dose (mg)	Administration frequency	Administration days
dose regimen used in clinical trials	200	Q12 h	7
tested dose regimen	400	Q24 h	7

**TABLE 3:** Simulation of TDI01 PK parameters.

Dosing regimens	Steady-state PK parameter	Median	95% confidence intervals (CI)
400 mg QD, 7 days	C_max,7d_ (ng/mL)	409.11	197.51–884.54
	AUC_0–24h, 7d_ (ng/mL·h)	4,939.64	1955.47–11888.33
200 mg BID, 7 days	C_max,7d_ (ng/mL)	466.18	190.54–1,068.16
	AUC_0–24h, 7d_ (ng/mL·h)	6,078.02	2,427.95–14598.77

## Discussion

As shown in the previous clinical study, the double peak phenomenon could be observed after single administration of TDI01. The simulation results suggested blood concentrations after continuous administration of TID01 are less than the C_max_ of 1,391 ng/mL under the highest single dose (1,200 mg) in clinical trial, which indicated the drug accumulation caused by hepato-enteral circulation would not affect the safety of TID01 usage under the tested multiple dosage regimens. The characteristic of multiple releases with time-dependent effects after a single dose indicated that hepato-enteral circulation model should be included in the PK model of TDI01 to describe its process *in vivo*.

As shown in Figure 1, the analysis results of blood drug concentration after dose normalization suggested that bioavailability in low dose groups (200 mg and 400 mg) was higher than that in high dose groups (800 mg and 1,200 mg), which need more clinical evidence to support this conclusion. The results of external validation indicated that the established models exert acceptable prediction capability on the plasma concentration-time profiles of TDI01 after single- and multiple-dose, but there are some bias in predicting the high concentrations (Figures 4, 5).

Our study has several limitations. The data used to develop the Pop-PK models were derived from 39 healthy subjects. The restricted sample size and homogeneous demographic profile may not represent the broader patient population, and potentially overlooked critical covariates affecting PK characters. Therefore, further empirical evidence is necessary to validate the proposed PK models. Patient studies, particularly the patients suffering different severities of ALI/ARDS, and multi-center trials may be used to address the limitations of the current work.

The covariate model was not developed in this study for the following reasons: (1) The PK data was obtained from healthy subjects, and their demographic and physiological characteristics were similar, so it is difficult to select significant covariates; (2) The purpose of this study was to evaluate the *in vivo* exposure level of TDI01 in the subjects after single- and multiple-dose through simulation.

## Conclusion

In summary, the proposed Pop-PK model allowed a thorough interpretation of the pharmacokinetic character of TDI01. Additionally, the established hepato-enteral circulation model can be used to fit the double peak phenomenon induced by the hepato-enteral circulation. Furthermore, the model-based simulation may be helpful in guiding the choice of dosage regimens for TDI01, eventually assist in assuring the clinical medication safety and maximizing therapeutic efficacy.

## Data Availability

The raw data supporting the conclusions of this article will be made available by the authors, without undue reservation.

## Abbreviations

NONMEM, nonlinear mixed-effect modeling; Pop-PK, Population pharmacokinetic; VPC, visual predictive check; CWRES, Conditional weighted residuals; FOCEI, first-order conditional estimation with interaction; OFV, Objective function value; AIC, Akaike information criterion.
